# Differences in Itch Quality Between Interleukin-31 and Thymus and Activation-Regulated Chemokine of Japanese Atopic Dermatitis

**DOI:** 10.1016/j.mayocpiqo.2026.100695

**Published:** 2026-02-20

**Authors:** Yozo Ishiuji, Minako Ogawa-Tominaga, Michie Katsuta, Itaru Dekio, Shiho Kawagoe, Yoko Kobayashi, Yoshimasa Nobeyama, Budiman Kharma, Hiroshi Kawasaki, Toshiya Ebata, Motoki Morita, Mitsutoshi Tominaga, Kenji Takamori, Akihiko Asahina

**Affiliations:** a Department of Dermatology, The Jikei University School of Medicine, Tokyo, Japan; b Laboratory for Developmental Genetics, RIKEN Center for Integrative Medical Sciences, Yokohama, Japan; c Chitofuna Dermatology Clinic, Tokyo, Japan; d Juntendo Itch Research Center, Institute for Environmental and Gender-Specific Medicine, Juntendo University Graduate School of Medicine, Chiba, Japan; e Department of Dermatology, Juntendo University Urayasu Hospital, Chiba, Japan

## Abstract

**Objective:**

To elucidate the relationships among various itch qualities, blood biomarkers, and patient-reported outcome measures (PROMs) in atopic dermatitis (AD), we characterized 10 qualities of itch (QoIs) in adult Japanese patients with AD and examined their associations with blood biomarkers as well as objective and subjective clinical measures.

**Patients and Methods:**

The QoI was assessed by a translated 10-item questionnaire. The relationship among each QoI and blood biomarkers (including interleukin [IL]-31, thymus and activation-regulated chemokine [TARC], and lactate dehydrogenase levels; differential white blood cell counts, including eosinophil, basophil, and neutrophil relative counts; and total immunoglobulin E levels); PROMs, such as visual analog scale and patient-oriented eczema measure; and objective measures, such as eczema area and severity index, was evaluated.

**Results:**

Patients with AD frequently suffered from a sensation similar to crawling like ant (crawling) and stinging and burning sensations. Crawling was positively correlated with serum IL-31 levels but not serum TARC level. Stinging and stabbing sensations were positively correlated with serum TARC level but not with serum IL-31 levels. Stinging, stabbing, and burning sensations were correlated with PROMs and eczema area and severity index. Our limitation is that other cytokines, such as IL-4 and IL-13, were not measured.

**Conclusion:**

The QoI in Japanese patients with AD could be classified into IL-31-related and TARC-related types. Our findings suggest relationships between crawling and IL-31 and between stinging and stabbing and TARC in Japanese patients with AD.

Itch is the cardinal symptom of atopic dermatitis (AD).^1^ Itch has multiple aspects, including a sensory aspect, such as recognizing the intensity and location of the itch; an emotional aspect, such as the unpleasant and pleasant sensations associated with scratching; and a motivational aspect, which generates the desire to scratch.^2^

In recent years, research advances have led to the emergence of new treatments for itch, including several biological inhibitors and Janus kinase inhibitors. It is important to assess itch accurately to select the appropriate itch treatment. The evaluation of itch is challenging due to the involvement of various factors. The major evaluation method is based on intensity, which is evaluated using the visual analog scale (VAS) and numerical rating scale. The use of peak numerical rating scale is recommended by the harmonizing outcome measures for eczema initiative.^3^ However, itch is not an isolated symptom, and different types exist. Quality of itch (QoI) differs according to the disease,^4^ with AD, psoriasis, and urticaria having different QoIs.^5^ Cowhage-induced itch is often pricking, whereas the sensation induced by capsaicin is often felt as burning.^6^ Moreover, burning has been reported to be common in European patients with AD,^5^ whereas the sensation of crawling like ants (crawling) has been reported to be common in Chinese patients with AD.^7^ Asking questions about the quality of pain can lead to accurate judgment of the type of pain and understanding of its pathological condition, which are useful when choosing an appropriate treatment. Serum interleukin (IL)-31, thymus and activation-regulated chemokine (TARC), and lactate dehydrogenase (LDH) are known biomarkers of the intensity of itch. The relationships among QoI, serum biomarkers, and objective and subjective assessments in AD remain to be elucidated.

The aim of this study was to evaluate itch from various aspects, focusing on QoI and its relationship to various serum biomarkers, including IL-31 and TARC. In addition, the association of QoI with patient-reported outcome measures (PROMs), such as VAS and patient-oriented eczema measure (POEM), and objective measures, such as eczema area and severity index (EASI), was also investigated. If these associations are confirmed, it may be possible to predict the levels of relevant serum biomarkers and the results of other PROMs without the need for blood sampling.

## Patients and Methods

### Patients

The Ethics Committee of the Jikei University School of Medicine (Tokyo, Japan) approved the study protocol (approval number # 25-210 & 27-298). All patients provided written informed consent before enrollment. For this study, 132 patients with AD were recruited (94 men and 38 women) who met the following inclusion criteria: (i) referral to the Jikei University School of Medicine; (ii) fulfillment of the diagnostic criteria for AD described by Hanifin and Rajka^8^; and (iii) agreement to assessment of subjective severity of AD by VAS for itch, POEM, 5D itch scale, and EASI, and to assessment of objective severity of AD by peripheral blood tests measuring IL-31, TARC, LDH levels, and differential white blood cell counts, including eosinophil, basophil, and neutrophil relative counts, as well as total immunoglobulin E (IgE) levels. The mean age of recruited patients was 44.4 years (range 18-80 years). Exclusion criteria were: (i) administration of systemic immunosuppressants, systemic corticosteroids, biologics, or Janus kinase inhibitors; (ii) undergoing treatment with phototherapy; and (iii) pregnant or breast-feeding.

### Itch Assessments

Various QoIs were evaluated by translating Yosipovitch et al^7^ questionnaire into Japanese, with a total of 10 items. The characteristics of itch sensation were assessed by sensory and affective dimensions. The sensory description of sensation of itch was constructed from a list of 6 words: tickling, stinging, crawling, stabbing, pinching, and burning. The affective dimension was composed of a set of 4 words commonly mentioned by patients suffering from itch: bothersome, annoying, unbearable, and worrisome. The intensity of itch and pain was assessed by VAS. Itch-related ratings were evaluated using 5D scores and POEM. The quality of life was assessed by dermatology life quality index (DLQI).

### Disease Severity

Subjective severity was scored by Japanese Dermatological Association-authorized dermatologists and patients. The segment score of EASI for erythema was calculated as the total sum of scores of erythema multiplied by both the area score and 0.1 for head/neck, 0.3 for trunk, 0.2 for upper limbs, and 0.4 for lower limbs, which are constants that reflect the relative contribution of those regions to the total body surface area. Segment scores of EASI for induration/papulation, excoriation, and lichenification were calculated as total sums and multiplied by both area score and 0.1 for head/neck, 0.3 for trunk, 0.2 for upper limbs, and 0.4 for lower limbs.

### Serum Examination

Differential white blood cell count was performed with an automatic blood cell count analyzer (XE-5000; Sysmex). Biochemical blood analysis was performed with an automatic immunity analyzer (HISCL-5000; Sysmex) for TARC level, automatic analyzer (LABOSPECT 008α; Hitachi) for LDH level, and automatic chemiluminescent enzyme immunity analyzer (AIA-CL2400; Tosoh) for total IgE level. The association between multifactorial factors of itch and serum biomarkers was evaluated. Interleukin-31 level was determined by Human IL-31 DuoSet ELISA (R&D SYSTEMS), according to the manufacturers’ protocols.

### Statistical Analyses

Statistical analysis was performed using Prism version 9.5.0 software (GraphPad Software). Welch’s *t*-test was performed to evaluate differences in quantitative and qualitative values. Values of *P*<.05 were significant.

## Results

### 1. QoI in Japanese Patients With AD

**Figure 1:**
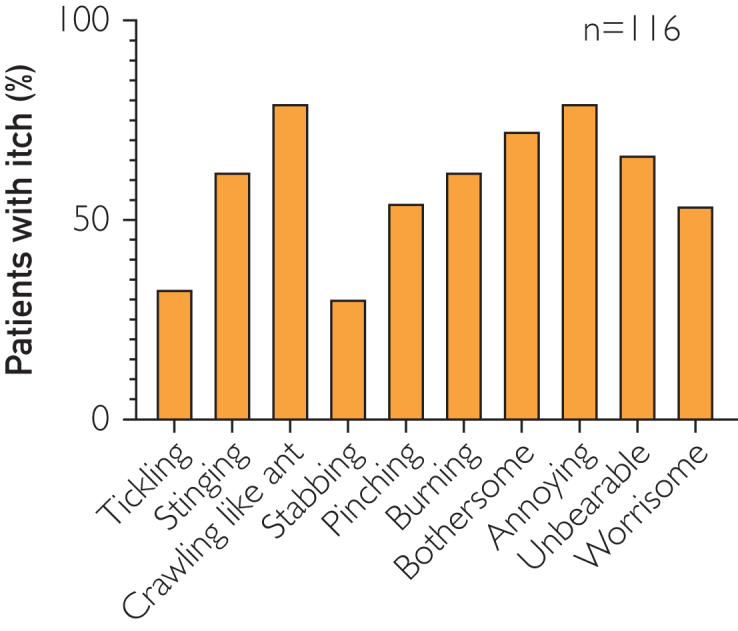
QoI in Japanese patients with AD. Percentage of patients with each QoI. AD, atopic dermatitis; QoI, quality of itch.

In terms of the sensory aspect of QoI, patients complained of crawling, stinging, and burning in that order. In terms of the affective aspect, patients complained of annoying, bothersome, unbearable, and worrisome in that order. Patients with AD were less likely to complain of stabbing and tickling (Figure 1).

### 2. Relationships Among QoI and Serum Factor Levels

**Figure 2:**
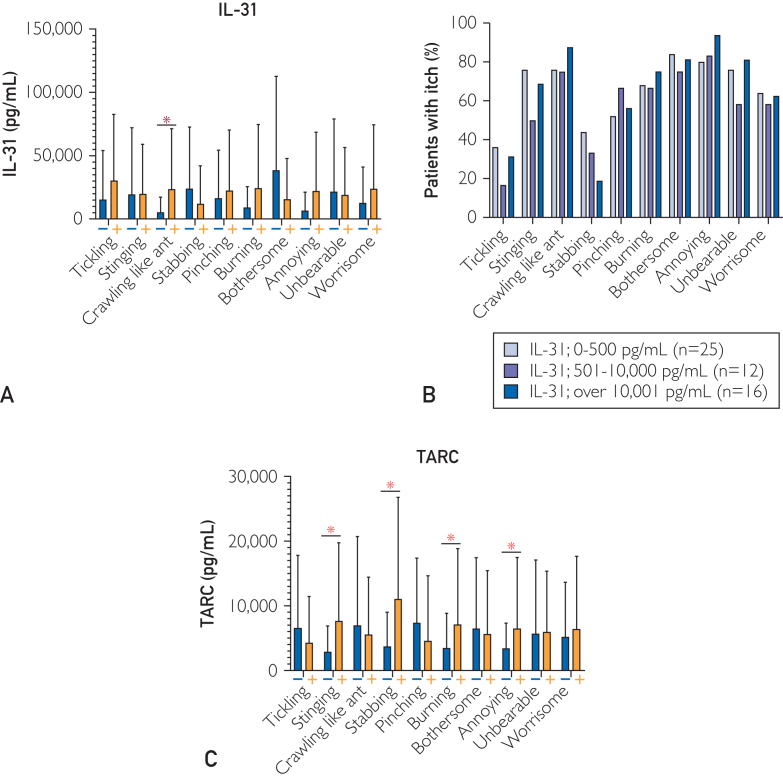
Relationship between QoI and IL-31 and TARC in Japanese patients with AD. (A) Asterisk indicates statistical significance. Serum IL-31 level in patients with and without each QoI. Serum IL-31 level was significantly higher in patients with crawling (*P*=.029) sensation. (B) Percentage of patients with each QoI in each patient group with serum IL-31 levels of under 500, 500 to 10,000, and over 10,000 pg/mL. (C) Asterisk indicates statistical significance. Serum TARC level in patients with and without each QoI. Serum TARC level was significantly higher in patients with stinging (*P*=.004), stabbing (*P*=.017), burning (*P*=.031), and annoying (*P*=.041). AD, atopic dermatitis; IL, interleukin; QoI, quality of itch; TARC, thymus and activation-regulated chemokine.

**Figure 3:**
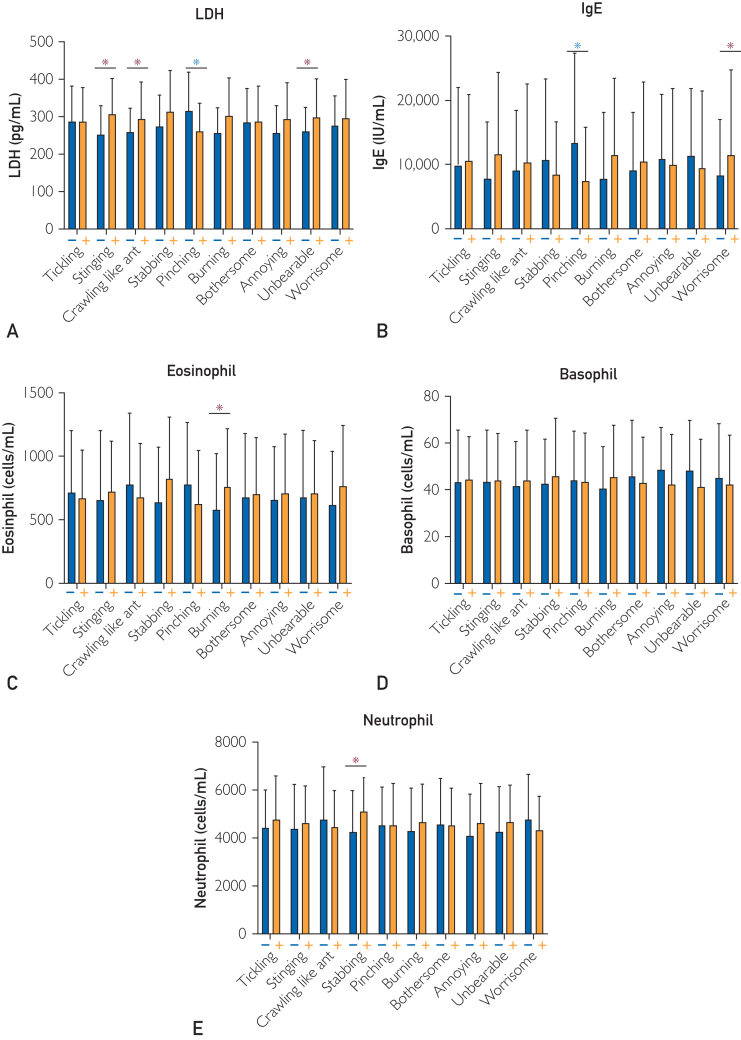
Relationship between QoI and each serum factor in Japanese patients with AD. (A) Serum LDH levels in patients with and without each QoI. (B) Serum IgE levels in patients with and without each QoI. (C) Eosinophil count in patients with and without each QoI. (D) Basophil count in patients with and without each QoI. (E) Neutrophil count in patients with and without each QoI. AD, atopic dermatitis; IgE, immunoglobulin E; IL, interleukin; LDH, lactate dehydrogenase; QoI, quality of itch. ∗*P*<.05 (Student *t* test).

We examined correlations among each QoI and each serum factor. Serum IL-31 level was significantly higher in patients with crawling (*P*=.029) sensation. Furthermore, the level was higher in patients with tickling, burning, annoying, and worrisome, and lower in patients with stabbing (Figure 2A). In particular, serum IL-31 concentration was inversely correlated with stabbing in a concentration-dependent manner (Figure 2B). Serum TARC level was significantly higher in patients with stinging (*P*=.004), stabbing (*P*=.017), burning (*P*=.031), and annoying (*P*=.041). The level was higher in patients with unbearable and worrisome sensations, and lower in those with tickling, crawling, pinching, and bothersome sensations (Figure 2C). Serum LDH level was significantly higher in patients with stinging (*P*=.003), crawling (*P*=.048), burning (*P*=.006) and unbearable (*P*=.005), and significantly lower in those with pinching (*P*=.002) (Figure 3A). Serum IgE level was significantly higher and lower in patients with worrisome (*P*=.002) and pinching (*P*=.013), respectively (Figure 3B). Eosinophil count was significantly higher in patients with burning (*P*=.050) (Figure 3C). Basophil count was higher in patients with tickling, stinging, crawling, stabbing, and burning, and lower in those with pinching, bothersome, annoying, unbearable, and worrisome sensations (Figure 3D). Neutrophil count was significantly higher in patients with stabbing (*P*=.010) (Figure 3E).

### 3. Classification of QoI Based on Blood Factor Patterns

**Figure 4:**
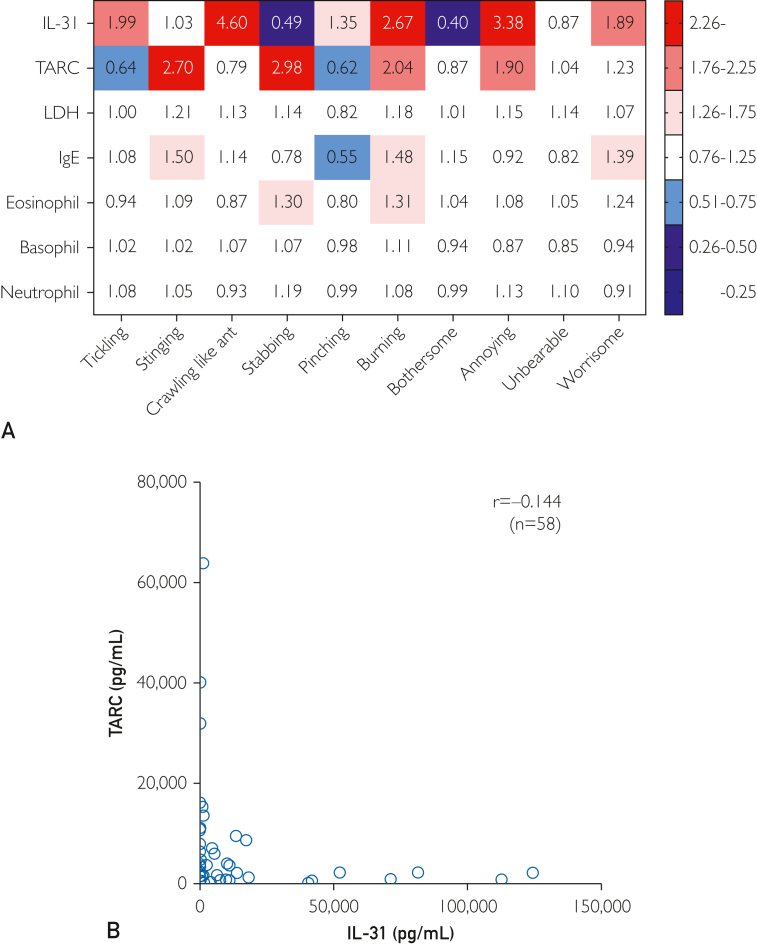
Classification of QoI based on the expression patterns of blood factors. (A) Correlation between serum IL-31 and TARC levels. Difference of QoI pattern between serum IL-31 and TARC levels. Percentage change was calculated by dividing the number of people in the group with each QoI by the group without it. We classified 0.25 and below as strongly negative, 0.26-0.50 as moderately negative, 0.51-0.75 as mildly negative, 0.76-1.25 as invariant, 1.26-1.75 as mildly positive, 1.76-2.25 as moderately positive, and 2.26 and above as strongly positive. (B) The correlation between serum IL-31 and serum TARC levels. There were few patients with elevated levels of both serum IL-31 and TARC. The correlation between serum IL-31 and serum TARC levels was analyzed. We identified a trend toward an inverse correlation between IL-31 and TARC in blood; however, the finding was not significant. IgE, immunoglobulin E; IL, interleukin; LDH, lactate dehydrogenase; QoI, quality of itch; TARC, thymus and activation-regulated chemokine.

We further examined percentage changes in each blood biomarker in the groups with and without each QoI. From the results, a heatmap was generated, which was used to define 0.25 and below as strongly negative, 0.26-0.50 as moderately negative, 0.51-0.75 as mildly negative, 0.76-1.25 as invariant, 1.26-1.75 as mildly positive, 1.76-2.25 as moderately positive, and 2.26 and above as strongly positive (Figure 4A).

We identified correlations between the QoI pattern match rate (%) and serum IL-31/TARC for each of the 15 patterns generated in Supplemental Figure 1B (available online at http://www.mcpiqojournal.org). The heat map was revised based on the high correlation coefficient between serum IL-31/TARC and the positive/negative pattern matching rate of each QoI (Supplemental Figure 1B and C). Pattern No. 1 showed the highest positive correlation between patient QoI pattern concordance rate and serum IL-31. The QoI pattern that showed the highest positive correlation with serum TARC was pattern No. 8. From these analyses, positive and negative patterns for each QoI were created for IL-31 and TARC, and the best-fit pattern was defined as itch quality associated with IL-31 and TARC (Supplemental Figure. 1C). The concordance rates between the IL-31–dominant and TARC-dominant patterns are presented in Supplemental Figure 1E and F and Table.

Interleukin-31 was strongly positive for crawling, burning, and annoying; moderately positive for tickling and worrisome; mildly positive for pinching; and negative for stabbing and bothersome. On the contrary, TARC was strongly positive for stinging and stabbing, moderately positive for burning and annoying, and negative for tickling and pinching (Figure 4A).

**Figure 5:**
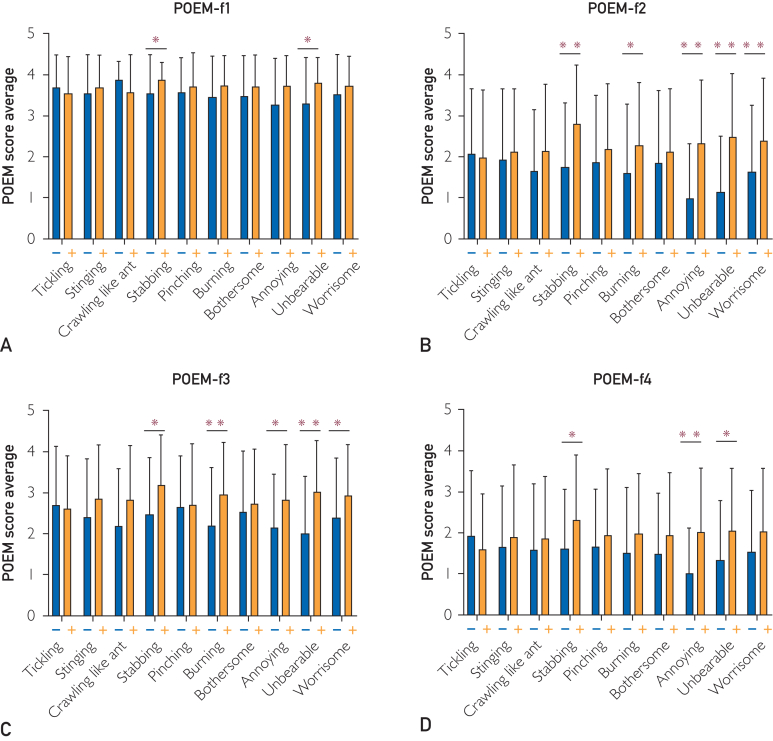

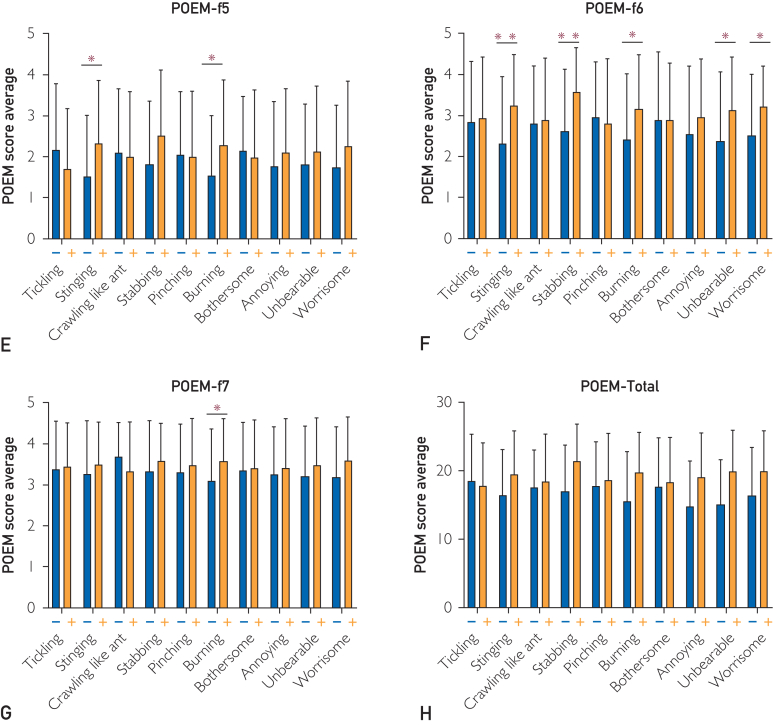
Relationship among each QoI and each portion of POEM. (A) Itch (f1) score in patients with and without each QoI. (B) Sleep (f2) score in patients with and without each QoI. (C) Bleeding (f3) score in patients with and without each QoI. (D) Weeping or oozing clear fluid (f4) score in patients with and without each QoI. (E) Cracked (f5) score in patients with and without each QoI. (F) Flaking off (f6) score in patients with and without each QoI. (G) Dryness (f7) score in patients with and without each QoI. (H) Total POEM score of patients with and without each QoI. Referring to each portion of POEM, the score of itch (f1) was significantly higher in patients with stabbing (*P*=.021), and unbearable (*P*=.001). The score of sleep (f2) was significantly higher in patients with stabbing (*P*=.001), burning (*P*=.043), annoying (*P*<.001), unbearable (*P*<.001), and worrisome (*P*=.014) sensations. The score of bleeding (f3) was significantly higher in patients with stabbing (*P*=.012), burning (*P*=.006), annoying (*P*=.041), unbearable (*P*<.001), and worrisome (*P*=.046) sensations. The score of weeping or oozing clear fluid (f4) was significantly higher in patients with stabbing (*P*=.044), annoying (*P*=.001), and unbearable (*P*=.022) sensations. The score of cracked (f5) was significantly higher in patients with stinging (*P*=.010) and burning (*P*=.016) sensations. The score of flaking off (f6) was significantly higher in patients with stinging (*P*=.003), stabbing (*P*<.001), burning (*P*=.019), unbearable (*P*=.026), and worrisome (*P*=.016) sensations. The score of dryness (f7) was significantly higher in patients with burning (*P*=.044) sensations (Supplemental Figure 1A-G). Total POEM score was significantly higher in patients with stinging (*P*=.029), stabbing (*P*=.001), burning (*P*=.004), annoying (*P*=.010), unbearable (*P*<.001), and worrisome (*P*=.001) sensations. POEM, patient-oriented eczema measure; QoI, quality of itch. ∗*P*<.05, ∗∗*P*<.01 (Student *t* test).

We found that both IL-31 and TARC were positive for burning and annoying. Regarding other QoIs, tickling, crawling, pinching, and worrisome were only positive in IL-31, whereas bothersome was only negative in IL-31. On the contrary, stinging and stabbing were only positive in TARC. There were few patients with both elevated levels of serum IL-31 and TARC. Figure 5 shows correlations between serum IL-31 and serum TARC levels. These results suggest that QoI with the presence of tickling, crawling, pinching, and worrisome and the absence of stabbing and bothersome represents an IL-31-related itch, whereas QoI with the presence of both stinging and stabbing and the absence of tickling and pinching represents a TARC-related itch. The patterns of the correlations of TARC levels with each sensory QoI were similar to those of IgE level (Figure 4A).

### 4. Relationship Among Each QoI and Other PROMs

Patients with high itch intensity (itch VAS of 50 or higher) were more likely to complain of QoIs such as stinging, stabbing, burning, bothersome, annoying, unbearable, and worrisome. Patients with pinching reported lower scores in the scale (Supplemental Figure 2A, available online at http://www.mcpiqojournal.org). On the contrary, compared with itch, pain VAS was higher in patients with the following QoIs in terms of pain intensity: stinging, stabbing, pinching, burning, annoying, unbearable, and worrisome (Supplemental Figure 2B). The 5D itch score was higher in patients with any of the QoI components (Supplemental Figure 2C). The DLQI score was significantly higher in patients with stinging (*P=*.002), stabbing (*P*<.001), burning (*P*<.001), annoying (*P*<.001), unbearable (*P*<.001), and worrisome (*P*<.001) sensations (Supplemental Figure 2D). The score was higher in those with tickling, crawling, pinching, and bothersome sensations. The total score of POEM was higher in patients with stinging, crawling, stabbing, pinching, burning, bothersome, annoying, unbearable, and worrisome sensations, whereas it was lower in those with tickling (Figure 5). An increased proportion of each QoI correlating with higher severity of EASI was found in all QoIs except pinching and bothersome (Supplemental Figure 2E).

## Discussion

Our findings suggest that itch in patients with AD can be classified into IL-31-related and TARC-related itch using a QoI questionnaire. Correlation analyses between QoI and blood biomarkers showed that serum IL-31 level was positively associated with crawling, whereas it was negatively associated with stabbing (Figure 2A and B and Figure 4). We found that: (i) crawling was the most frequent QoI (Figure 1) and (ii) there was a strong positive correlation between blood IL-31 and the QoI of crawling. These findings suggest that crawling is the most common QoI and it is mediated by IL-31 in Japanese adult patients with AD. Serum TARC level was positively correlated with stinging, stabbing, and burning, and negatively correlated with tickling, crawling, and pinching (Figure 2C). On the contrary, serum IL-31 level was not positively correlated with stinging and stabbing (Figure 4A). There were few cases in which both IL-31 and TARC were elevated simultaneously, and there was a slight inverse association between IL-31 and TARC in blood (Figure 4B). These findings suggest that interviewing patients about crawling and stinging/stabbing sensations predicts whether the itch is IL-31-related or TARC-related, even without examining serum biomarkers. In cases where the interview is not enough to determine the type of itch, asking about a tickling sensation may improve accuracy.

Our findings suggest that it is possible to infer factors in the blood related to itch by asking questions about QoI, without the need for blood draws. Although related blood factors are not considered to be the complete cause of itch, taken together, QoI is a useful interview tool for formulating a treatment plan for patients with AD.

We found that crawling was the most common QoI in Japanese AD, and IL-31 is related to crawling. Interleukin-31 has been implicated in the pathophysiology of multiple atopic disorders, including AD, allergic rhinitis, and airway hyper-reactivity. In AD, IL-31 has been identified as one of the main drivers of its cardinal symptom, pruritus. TH2 cells play a central role in AD and release high levels of TH2-associated cytokines including IL-31, thereby mediating inflammatory responses, initiating immunoregulatory circuits, and stimulating itch and neuronal outgrowth through activation of the heterodimeric receptor IL-31 receptor A (IL-31RA)/oncostatin M receptor β. Interleukin 31RA expression is found on human and murine dorsal root ganglia neurons; epithelial cells, including keratinocytes; and various innate immune cells. Interleukin-31 is a critical cytokine involved in neuroimmune communication, suggesting the role of cytokine modulation in neuroinflammatory diseases, including AD/itch, which supports the findings of recent clinical trials using an anti-IL-31 antibody.^9^ Thus, inhibition of IL-31-downstream signaling may be a beneficial approach for various inflammatory diseases, including AD.^10^

A previous study reported that IL-31RA expressed by sensory neurons but not by keratinocytes was required for the itch induction by IL-31.^11^ This suggests that sensory neuronal IL-31RA and STAT3 have roles in IL-31-induced itch. Moreover, sensory neuronal STAT3 may contribute to IL-31-independent inflammatory itch. Our results suggest the involvement of IL-31 in noninflammatory, neuropathic itch, supporting a previous study.^12^

In addition to AD, IL-31 has been implicated in other skin diseases, including psoriasis,^13^ prurigo nodularis,^14^ and chronic pruritus of unknown origin^15^ as well as systemic diseases involving chronic liver dysfunction^16^ such as primary biliary cirrhosis (PBC) and chronic-kidney-disease-associated pruritus.^17^ Among them, baseline serum IL-31 levels in PBC were positively correlated with VAS for pruritus and 5-D itch scores.^16^ The IL-31 levels are correlated with pruritus in patients with cholestatic and metabolic liver diseases and are farnesoid X receptor responsive in nonalcoholic steatohepatitis.^16^ In addition, patients with PBC described their itch as bugs crawling.^18^ These results suggests that crawling is an important QoI for Asian AD and PBC. Therefore, the involvement of IL-31, which is a common feature of both diseases, may be the cause of the QoI of crawling in these diseases.

This study reported for the first time that stinging, stabbing, and burning sensations were strongly correlated with higher levels of serum TARC and higher scores of EASI and PROMs such as POEM and pain VAS (Supplemental Figure 2E).

Thymus and activation-regulated chemokine/CCL17 was reported to be the most reliable AD biomarker.^19^ As TARC/CCL17 is strongly correlated with AD disease severity, its levels are used to assess therapy efficacy.^20^ In Japan, TARC/CCL17 has been measured commercially for health insurance purposes since 2008. This may be reflected in the quality of TARC/CCL17-related itching and its association with skin inflammation and dryness. According to POEM, among the QoIs, cracked skin was strongly associated with stinging and burning; flaking off was associated with stinging, stabbing, burning, and dryness sensation; and rough skin was associated with burning. Items 5 to 7 of POEM represent the degree of skin inflammation and dryness. The QoIs, including stinging, stabbing, and burning, are all related to TARC/CCL17 and inflammation symptoms such as skin dryness. Thus, certain QoIs, such as stinging, stabbing, and burning, may be useful as indicators of the disease severity of AD. Stinging and burning are important QoIs for inflammatory itch.^21^

Stinging, stabbing, burning are important for itch VAS (Supplemental Figure 2A). Stinging and stabbing are also important for pain VAS (Supplemental Figure 2B). In addition, pain VAS is also associated with itch VAS and EASI (Supplemental Figure 3A and B, available online at http://www.mcpiqojournal.org). As these QoIs are TARC-related, TARC-related QoI and intensity of pain are important for assessing the intensity of itch. Furthermore, itch VAS scores were not correlated with peripheral blood eosinophil, basophil, or neutrophil counts (Supplemental Figure 4).

In animal models of inflammatory arthritis, including zymosan-induced and antigen-induced arthritis, TARC/CCL17 gene-deficient mice fail to develop arthritic pain and optimal disease. Additionally, the therapeutic administration of anti-mouse TARC/CCL17 monoclonal antibodies ameliorates already established arthritic pain and halts disease progression.^22^^,^^23^ Increased TARC/CCL17 expression is seen in the dorsal root ganglia of rats induced with chronic neuropathic pain, and intrathecal administration of TARC/CCL17 led to heightened thermal hypersensitivity and pronociception.^24^ These studies suggest the role of TARC/CCL17 in the development and progression of inflammatory arthritic pain.

A previous study reported that QoIs, such as stinging, burning, sunburn-like sensation, pain, and hurting, are more evident in inflammatory forms of itch.^21^ Considering these previous reports and our current data regarding TARC, TARC-related QoI may be related not only to dermatitis and dryness but also to pain.

Previously, Darsow et al^25^ revised McGill’s pain questionnaire and investigated the presence of 80 types of itch. Hawro et al^6^ reported that the quality of histamine-indued itch differs from that of chloroquine-induced itch. Brenaut et al^4^ suggested that QoI varies among diseases. In that context, Yosipovitch et al^7^ reported that crawling was most common among QoI in Chinese patients with AD, followed by tickling, both of which appeared more commonly than burning or stinging sensations.^7^ A study from Europe reported that stinging and burning more commonly appeared in AD.^5^ This study found that most Japanese patients with AD suffer from crawling, stinging, and burning sensations among sensory aspects of QoI (Figure 1). Thus, our findings support the hypothesis that there may be a racial component to the main QoIs in AD.

The reason for this discrepancy may be the difference in the subset of T cells responsible for AD due to racial differences. In AD, Th2, Th22, Th17, and Th1 play important roles in acute and chronic phases.^26^ Asian patients have accentuated polarity of the Th22/Th17 pathways, and also exhibit epidermal barrier defects despite relative maintenance of filaggrin and loricrin expression.^27^ Various type 2 cytokines, such as IL-31, IL-4, and IL-13, induce itch; however, IL-17A does not induce itch directly^28^ and has been reported to be associated with pain.^29^ In patients with AD, IL-17A may induce itch rather than pain due to a pruritic hypersensitivity state known as alloknesis. Thus, QoI may differ depending on the cytokines secreted by the helper T subset involved and vary between races.

## Conclusion

In conclusion, this study found meaningful relationships among QoI; blood biomarkers, mainly L-31 and TARC; and PROMs such as POEM and DLQI. To distinguish between IL-31-related and TARC-related itch, the assessment of 5 QoIs (pinching, stinging, crawling, stabbing, and tickling) is important. Our results may be used to predict the efficacy of pruritus treatment; thus, further studies are needed to apply our findings to the selection of treatment options.

## Limitations

In AD, not only Th2 cytokines such as IL-4 and IL-13 but also the Th1, Th17, and Th22 pathways play important roles in the disease pathophysiology. Furthermore, activation of both the Th2 and Th17 pathways has been reported to be more prominent in Asian patients compared with those of European ancestry.^30^ Therefore, in addition to IL-31 and TARC, measurement of other cytokines—including IL-4 and IL-13—would have been desirable in this study. Although we attempted to measure these cytokines, their concentrations were below the limits of detection with the current assay methods. Further studies with improved detection techniques are warranted.

## Potential Competing Interests

Dr Ishiuji has received honoraria as a speaker from Maruho, Eli Lilly Japan, AbbVie, Otsuka, Sanofi, Regeneron, and grants from Hoshi Pharmaceutical. Dr Katsuta has received grants from Maruho. Dr Nobeyama has received grants from Taiho Pharma and Maruho. Dr Dekio received grants from KINS. Dr Kawasaki has received research funds (grants paid to his institution) from Torii. Dr Asahina has received honoraria as a speaker from Bristol Myers, Sun Pharma Japan, Janssen Pharma, Kyowa Kirin, UCB Japan and Eli Lilly Japan, and grants from Maruho. All other authors report that they have no conflict of interest to disclose.

## Ethics Statement

This study was conducted in accordance with the ethical standards of the institutional review board (or equivalent ethics committee) of The Jikei University School of Medicine (Approval No. [#25-210]) and with the principles of the Declaration of Helsinki. Written informed consent was obtained from all participants prior to their inclusion in the study.
