# Supplementary material for: Differences in Itch Quality Between Interleukin-31 and Thymus and Activation-Regulated Chemokine of Japanese Atopic Dermatitis
Source: Mayo Clin Proc Innov Qual Outcomes. 2026 Feb 20;10(2):100695. doi: 10.1016/j.mayocpiqo.2026.100695 (PMC12945512; doi:10.1016/j.mayocpiqo.2026.100695)
Supplement: Supplementary Material [file mmc1.pdf]

Supplemental Files

Supplemental Materials

Supplemental Figure legends

Supplemental Figure 1. Classification of QoI based on the several expression patterns of blood factors.

A: Correlations between the QoI pattern match rate (%) and serum IL-31/TARC for each of the 15 patterns generated in Supplemental Figure 1B are shown. Heat map devised based on the high correlation coefficient between serum IL-31/TARC and the positive/negative pattern matching rate of each QoI.

B: The correlation coefficient of r-value was calculated between each patient's concordance rate with the positive (red; +) and negative (blue; -) combination pattern for each QoI and the patient's serum IL-31 or TARC concentration. The QoI combination patterns with the highest r-value for each of IL-31 and TARC were extracted. *P*-values were indicated as the results of student *t* test,*; $P < .05$. For serum IL-31 and TARC associated with each QoI, the r-value of concordance rate was not high for the positive and negative patterns created only by the significant differences shown in No. 7 and No. 15, respectively. This suggests that the range of significant differences in rate of change alone was insufficient to more accurately determine the association between QoI and serum factors. This procedure was further performed for other patterns (No. 1-15 executed patterns are extracted in Supplemental Figure 2A and B) to identify positive and negative trends for IL-31 and TARC. Pattern No. 1 showed the highest positive correlation between patient QoI pattern concordance rate and serum IL-31. The QoI pattern that showed the highest positive correlation with serum TARC was pattern No. 8. We performed data analysis using the Python programming language in Jupyter Notebook and verified that No. 1 and No. 8 had the highest r values. Analysis included data preprocessing, exploratory data analysis, and statistical analysis using the Pandas library. The Python code used in the analysis is shown in Supplemental Figure 2D. Patterns No. 1 and No. 7 were adopted for IL-31 and TARC patterns (Supplemental Figure 1C). The respective concordance rates are shown in Supplemental Figure 1C.

C: As a result from Supplemental Figure 1A and B, the pattern shown in Supplemental Figure 1C had the highest concordance rate for IL-31 and TARC.

D: The Python code and data used in the analysis.

E: Concordance rates for the IL-31–dominant and TARC-dominant patterns stratified by sex.

F: Number and percentage of patients with IL-31-dominant QoI and TARC-dominant QoI. Individual IL-31–type match rates and TARC-type match rates are shown. Paired values from the same individual are connected by lines. Lines representing IL-31–dominant individuals are shown in red, those representing TARC-dominant individuals are shown in blue, and those representing neutral individuals are shown in gray. (Left) The y-axis indicates the percentage of patients (%), and the numbers within the stacked bars represent the actual number of patients. (Right)

Supplemental Table 1. Match rate for QoI (%)

Differences in agreement rates between males and females for each type were evaluated using the Mann–Whitney U test. The asterisk indicates statistical significance (*P*= .016).

Supplemental Figure 2. Relationship among each sensory QoI and other PROMs.

Asterisk indicates statistical significance. A: Percentage of the patients with each QoI in each patient group with itch VAS score of over 50 or under 49. B: Percentage of the patients with each QoI in each patient group with pain VAS score of over 50 or under 49. C: 5D itch scale score of the patients with and without each QoI. Stabbing; *P*<.001, Burning; *P*<.001, Annoying; *P*<.001, Unbearable; *P*<.001, Worrisome; *P*=.013. D: DLQI score of the patients with and without each QoI. Stinging; *P*=.002, Stabbing; *P*<.001, Burning; *P*<.001, Bothersome; *P*=.019, Annoying; *P*<.001, Unbearable; *P*<.001, Worrisome; *P*<.001. E: Percentage of the patients with each QoI in each group with EASI score of 0-19, 20-39, and 40-72.

Supplemental Figure 3. Correlation analysis among various pain VAS score, itch VAS score, and EASI. A weak positive correlation was found among itch VAS or EASI and pain VAS score (Supplemental Figure 3A, B).

Supplemental Figure 4. Correlation analysis between various serum biomarkers and itch VAS score. A weak positive correlation was found between eosinophil or neutrophil count and itch VAS score (Supplemental Figure 4A, C). No correlation was found between basophil count and itch VAS score (Supplemental Figure 4B).
