# Supplementary material for: Differences in Itch Quality Between Interleukin-31 and Thymus and Activation-Regulated Chemokine of Japanese Atopic Dermatitis
Source: Mayo Clin Proc Innov Qual Outcomes. 2026 Feb 20;10(2):100695. doi: 10.1016/j.mayocpiqo.2026.100695 (PMC12945512; doi:10.1016/j.mayocpiqo.2026.100695)

Supplemental Figure 1

A

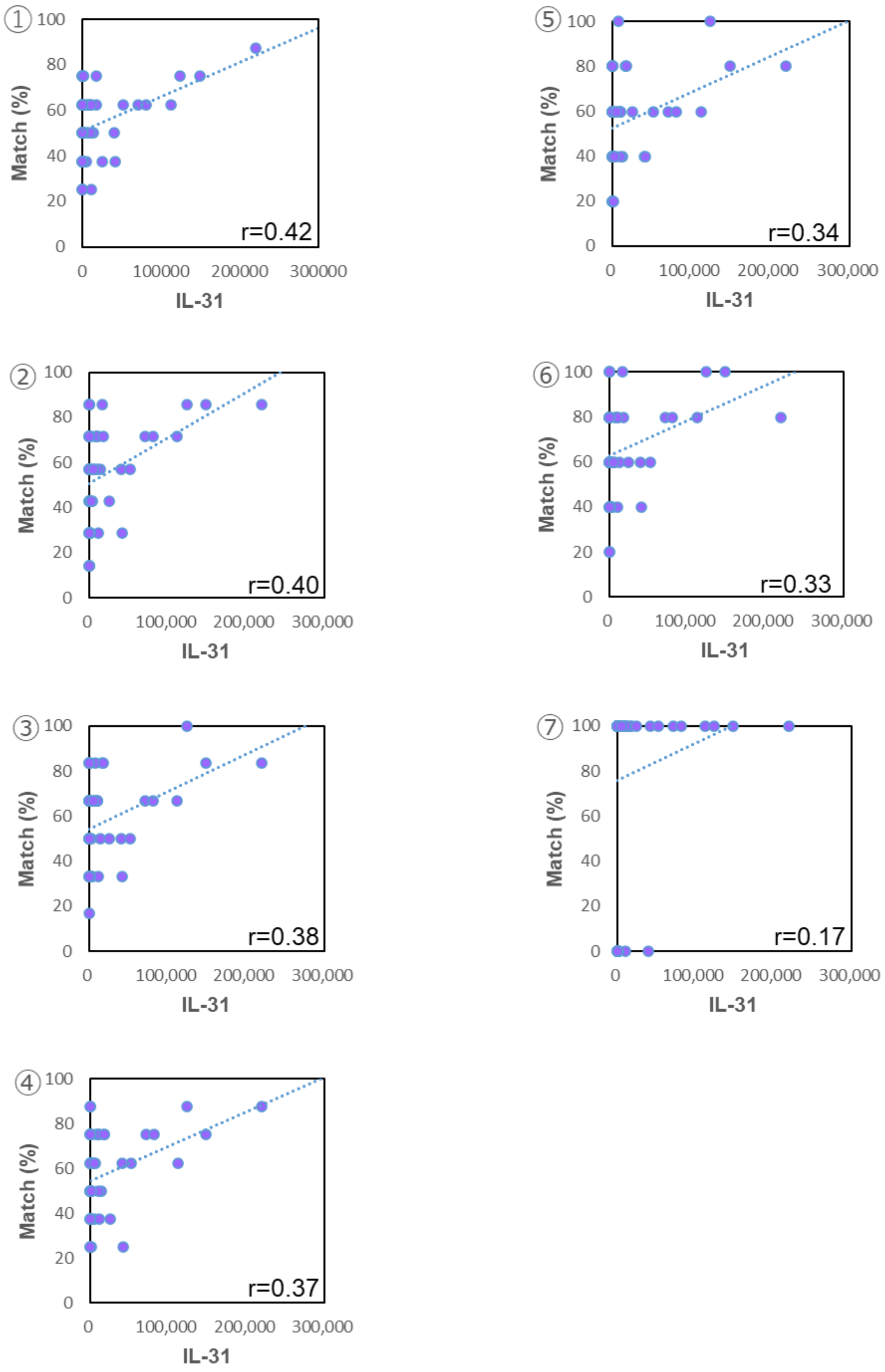

Supplemental Figure 1

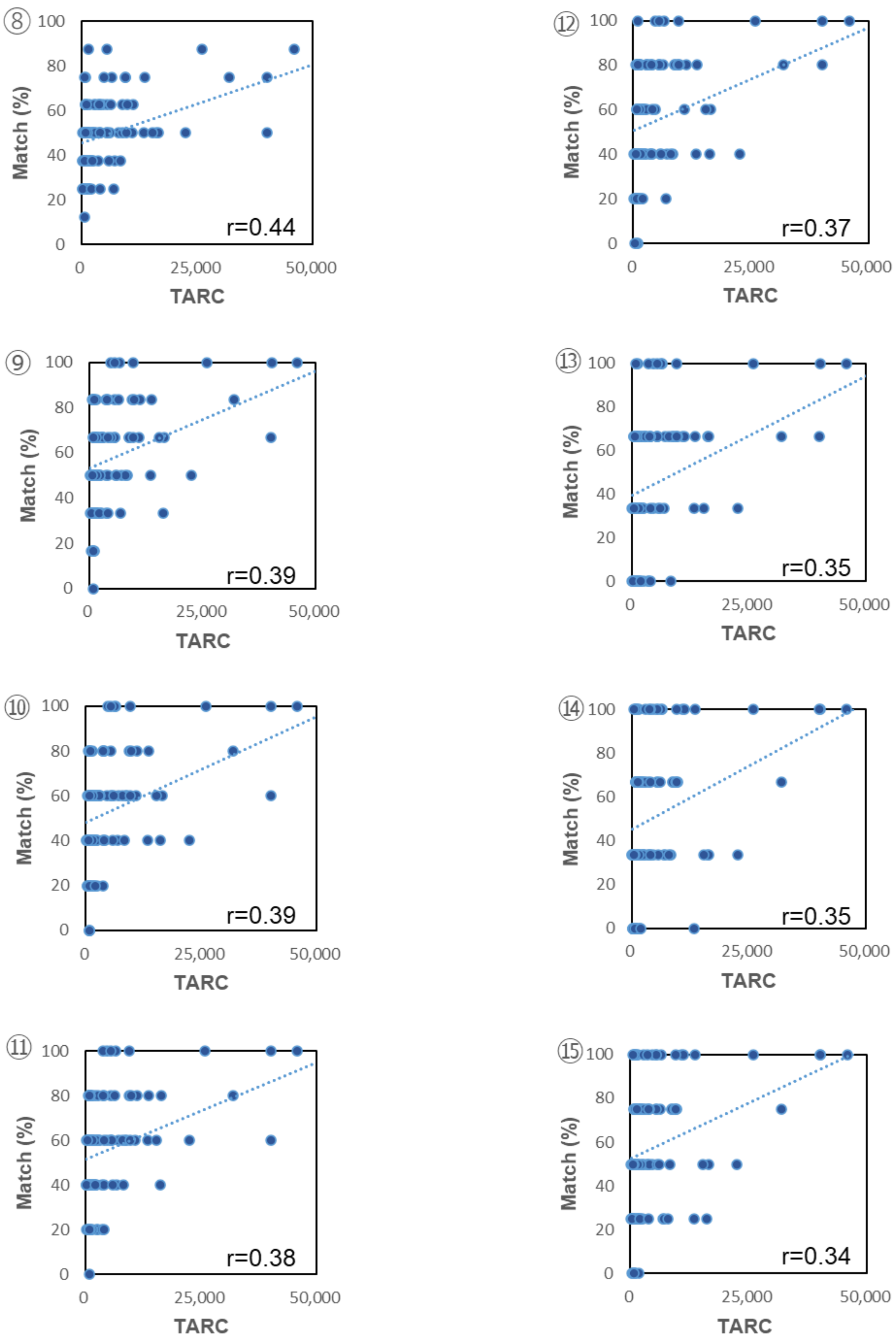

B

	QoI: tickling	stinging	crawling	stabbing	pinching	burning	bothersome	annoying	unbearable	worrisome	correlation coefficient (r=) with IL-31	correlation coefficient (r=) with TARC	
QoI combination selected based on the relationship between IL-31 and QoI (Fig. 2a)	+		+	-		+	-	+	-	+	① 0.42	0.00	
	+		+	-		+	-	+		+	② 0.40	-0.01	
	+		+	-		+	-	+			③ 0.38	-0.06	
	+		+	-	+	+	-	+		+	④ 0.37	-0.08	
	+		+	-			-	+			⑤ 0.34	-0.16	
	+		+	-		+		+			⑥ 0.33	-0.09	
			+								⑦ 0.17	-0.06	(i)
QoI combination selected based on the relationship between TARC and QoI (Fig. 3)	-	+	-	+	-	+	-	+			0.00	⑧ 0.44	
	-	+		+	-	+		+			0.03	⑨ 0.39	
	-	+		+	-	+					0.07	⑩ 0.39	
	+	+		+	-			+			0.11	⑪ 0.38	
		+		+	-	+		+			0.04	⑫ 0.37	
		+		+	-						0.10	⑬ 0.35	
		+		+		+					0.02	⑭ 0.35	
		+		+		+		+			0.08	⑮ 0.34	(ii)

(i) Combinations with significant differences in Fig. 2a
(ii) Combinations with significant differences in Fig. 3
①-⑮; Number in Supplementary Fig.S1a

C

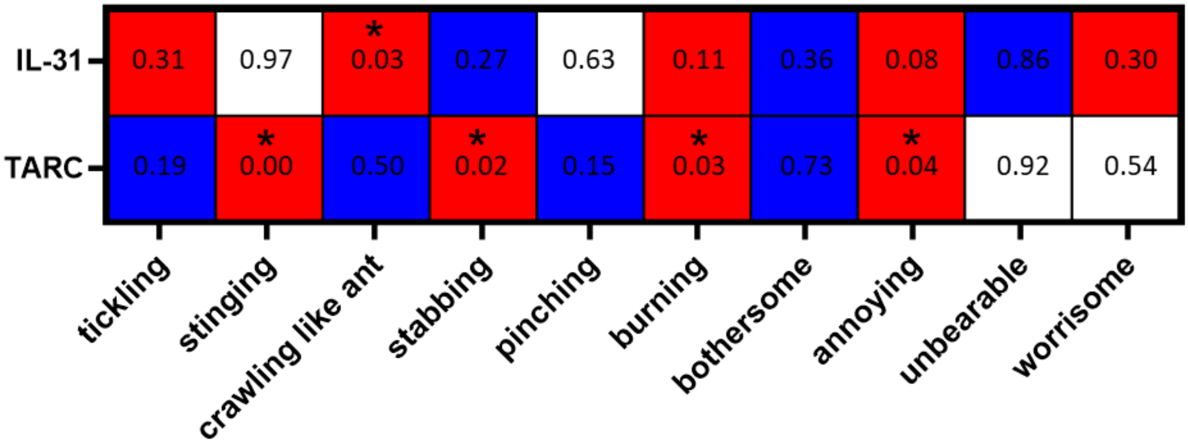

Supplemental Figure 1

D

```
In [1]:
import pandas as pd
In [2]:
file_path = 'C:¥¥Users¥¥moki¥¥Downloads¥¥Data file'   # shown below
In [3]:
df = pd.read_excel(file_path)
In [4]:
print(df.head())
 Pt.No.    A   B              α β γ δ ε ζ η θ ι κ      # A; TARC,  B; IL-31
0  Pt.1 1479.0 0.0      NaN 0 1 0 0 1 0 1 1 1 0
1  Pt.2 1854.0 0.0      NaN 1 1 1 0 1 1 1 1 1 1
2  Pt.3 2015.0 0.0      NaN 0 0 1 0 1 0 1 1 1 0
3  Pt.4 2994.0 0.0      NaN 1 1 1 0 0 1 1 1 1 0
4  Pt.5 3678.0 0.0      NaN 0 1 1 1 0 0 1 1 1 1
In [5]:
from itertools import combinations, product

def calculate_partial_match_rate(row, conditions):
    total_conditions = len(conditions)
    matches = sum(row[col] == val for col, val in conditions.items())
    match_rate = (matches / total_conditions) * 100
    return match_rate

def calculate_all_match_rates(df, columns):
    results = {}

    for num_items in range(1, len(columns) + 1):
        for combination in combinations(columns, num_items):
            for values in product([0, 1], repeat=num_items):
                condition = dict(zip(combination, values))
                condition_name = ', '.join(f"{k}={v}" for k, v in condition.items())
                results[condition_name] = df.apply(calculate_partial_match_rate, axis=1, conditions=condition)

    return pd.DataFrame(results)


columns_alpha_to_kappa = ['α', 'β', 'γ', 'δ', 'ε', 'ζ', 'η', 'θ', 'ι', 'κ']


all_match_rates = calculate_all_match_rates(df, columns_alpha_to_kappa)
In [6]:
def calculate_correlations_with_a_b(df, match_rates_df):   # a; TARC,  b; IL-31
    correlations_with_a = {}
    correlations_with_b = {}

    for condition in match_rates_df.columns:
        correlations_with_a[condition] = match_rates_df[condition].corr(df['A'])
        correlations_with_b[condition] = match_rates_df[condition].corr(df['B'])

    return correlations_with_a, correlations_with_b

correlations_with_a, correlations_with_b = calculate_correlations_with_a_b(df, all_match_rates)

max_correlation_with_a_condition = max(correlations_with_a, key=correlations_with_a.get)
max_correlation_with_a_value = correlations_with_a[max_correlation_with_a_condition]

max_correlation_with_b_condition = max(correlations_with_b, key=correlations_with_b.get)
max_correlation_with_b_value = correlations_with_b[max_correlation_with_b_condition]
In [7]:
print( max_correlation_with_a_condition, max_correlation_with_a_value)
print( max_correlation_with_b_condition, max_correlation_with_b_value)
α=0, β=1, γ=0, δ=1, ε=0, ζ=1, η=0, θ=1 0.437426210995885
α=1, γ=1, δ=0, ζ=1, η=0, θ=1, ι=0, κ=1 0.4186310780722708
```

Supplemental Figure 1

Data file

			tickling	stinging	crawling	stabbing	pinching	burning	bothersome	annoying	unbearable	worrisome
Pt.No.	A :TARC	B: IL31	α	β	γ	δ	ε	ζ	η	θ	ι	κ
Pt.1	1479	0	0	1	0	0	1	0	1	1	1	0
Pt.2	1854	0	1	1	1	0	1	1	1	1	1	1
Pt.3	2015	0	0	0	1	0	1	0	1	1	1	0
Pt.4	2994	0	1	1	1	0	0	1	1	1	1	0
Pt.5	3678	0	0	1	1	1	0	0	1	1	1	1
Pt.6	6545	0	0	1	1	1	0	1	1	1	1	1
Pt.7	10790	0	1	1	1	1	1	1	1	1	1	1
Pt.8	11170	0	0	1	1	1	1	1	1	1	1	1
Pt.9	3921	6.702	1	0	1	0	0	1	1	0	0	0
Pt.10	776	22.727	0	1	1	0	0	0	1	1	0	0
Pt.11	6332	50.64	0	1	1	1	0	1	1	0	1	0
Pt.12	16170	92.24	1	0	0	1	0	0	0	0	0	0
Pt.13	567	94.54	0	1	0	1	1	1	1	1	1	1
Pt.14	7965	110.2	0	1	0	0	0	0	1	0	1	1
Pt.15	1336	111.8	0	1	1	0	1	1	1	1	1	1
Pt.16	3926	112.7	0	1	1	1	1	1	1	0	0	0
Pt.17	4047	117	0	0	0	0	1	1	1	1	1	0
Pt.18	1272	130.5	1	1	1	1	0	0	1	1	1	1
Pt.19	40160	132.3	0	1	1	1	0	1	1	1	1	1
Pt.20	703	152	1	0	1	0	1	0	1	1	0	1
Pt.21	31900	157.8	0	1	1	0	0	1	0	1	1	1
Pt.22	859	230.5	1	1	1	0	1	1	1	1	1	1
Pt.23	503	244	0	1	1	0	1	0	0	1	0	0
Pt.24	691	269.5	1	0	0	0	1	1	1	1	1	1
Pt.25	4722	412.2	0	1	1	1	0	1	1	1	1	1
Pt.26	812	807.5	0	1	1	0	1	1	1	1	0	1
Pt.27	15360	868.256	0	0	1	0	0	1	1	1	1	0
Pt.28	330	1023	1	0	1	0	0	0	1	1	0	1
Pt.29	63880	1107	0	1	0	1	1	1	1	1	0	1
Pt.30	13590	1327	0	1	0	1	1	1	1	1	1	1
Pt.31	344	1508	0	0	0	0	0	0	1	1	0	0
Pt.32	3722	2616.714	0	1	1	1	1	1	1	1	1	1
Pt.33	7097	4494	0	1	1	0	0	0	1	0	1	1
Pt.34	5981	5453	0	1	1	0	1	1	0	0	0	0
Pt.35	1739	6501	0	0	1	1	1	1	0	1	1	0
Pt.36	671	7601	1	0	1	0	1	0	0	1	1	0
Pt.37	836	9839	0	0	1	0	1	1	1	1	1	1
Pt.38	4006	10260	0	1	1	0	0	1	1	1	1	0
Pt.39	3630	10980	0	0	1	0	1	1	1	1	1	1
Pt.40	717	11170	0	0	0	0	1	0	1	1	1	0
Pt.41	9512	13590	0	1	1	1	0	1	1	1	1	1
Pt.42	8692	17310	1	1	1	0	0	1	1	1	1	1
Pt.43	1229	18240	0	0	1	0	1	1	0	1	1	0
Pt.44		25180	0	1	1	0	0	0	1	1	1	0
Pt.45	135	40500	0	0	0	0	1	1	1	1	1	1
Pt.46	568	41930	0	1	1	0	0	0	1	0	0	0
Pt.47	2269	52410	0	1	1	0	1	0	1	1	0	1
Pt.48	942	71410	1	1	1	1	1	1	1	1	1	1
Pt.49	2292	81490	0	1	1	0	1	1	1	1	1	1
Pt.50	856	112700	1	1	1	1	0	1	1	1	1	1
Pt.51	2202	124500	1	1	1	0	1	1	0	1	1	0
Pt.52	5413	148800	1	1	1	0	0	1	1	1	1	1
Pt.53	556	220200	0	0	1	0	1	1	0	1	0	1
Pt.54	144		0	1	1	0	1	0	0	0	0	0
Pt.55	253		0	1	1	0	1	0	1	1	0	0
Pt.56	266		0	0	1	0	1	0	1	1	1	1
Pt.57	271		0	0	0	0	1	0	1	0	1	0
Pt.58	388		0	0	0	1	0	0	1	0	1	1
Pt.59	534		0	0	1	0	0	0	0	0	1	0
Pt.60	672		1	0	0	0	0	1	1	1	1	0

Supplemental Figure 1

				tickling	stinging	crawling	stabbing	pinching	burning	bothersome	annoying	unbearable	worrisome
Pt.No.	A :TARC	B: IL31		α	β	γ	δ	ε	ζ	η	θ	ι	κ
Pt.61	759			0	1	1	0	1	0	1	1	1	1
Pt.62	794			1	0	1	0	0	0	0	0	1	0
Pt.63	796			1	0	0	0	1	0	0	0	0	0
Pt.64	823			0	1	1	1	1	1	1	1	1	1
Pt.65	851			0	1	1	0	0	1	1	1	1	0
Pt.66	855			0	1	1	0	0	1	0	1	0	0
Pt.67	906			0	0	1	0	0	0	0	0	0	0
Pt.68	945			1	1	1	1	1	1	1	1	1	1
Pt.69	970			0	0	0	0	1	0	0	0	0	0
Pt.70	1027			0	1	1	0	0	1	1	1	1	0
Pt.71	1139			1	0	1	0	1	1	1	1	0	1
Pt.72	1341			0	0	1	0	1	0	1	1	0	0
Pt.73	1374			1	1	1	1	1	1	1	1	1	1
Pt.74	1398			0	1	0	0	0	1	0	1	0	0
Pt.75	1400			0	0	1	0	1	1	0	0	0	1
Pt.76	1419			1	0	1	0	0	1	1	1	1	0
Pt.77	1479			1	1	1	1	1	1	1	1	1	1
Pt.78	1610			0	1	1	0	1	1	1	0	1	1
Pt.79	1649			0	0	1	0	1	1	1	1	1	1
Pt.80	1659			0	0	1	0	1	0	1	1	0	0
Pt.81	1721			1	0	1	0	0	0	1	1	0	1
Pt.82	2101			1	1	1	0	1	1	1	1	1	1
Pt.83	2150			0	1	1	0	0	0	1	0	0	1
Pt.84	2160			0	1	1	1	1	0	1	1	1	1
Pt.85	2518			1	0	0	0	1	1	1	1	1	1
Pt.86	2548			1	0	0	0	0	1	0	0	0	0
Pt.87	2747			1	1	1	0	0	1	0	1	0	0
Pt.88	2827			0	1	1	0	0	0	0	1	1	0
Pt.89	2875			1	1	1	1	1	1	1	1	1	1
Pt.90	3654			1	0	0	0	1	1	0	1	1	1
Pt.91	4577			0	0	1	0	0	1	0	1	1	1
Pt.92	5425			0	1	0	1	0	1	1	1	0	0
Pt.93	5435			0	1	1	0	0	1	1	1	1	1
Pt.94	5524			1	1	1	1	1	1	1	1	0	0
Pt.95	5753			0	1	1	0	1	0	1	1	1	1
Pt.96	6829			0	1	1	0	1	0	1	0	1	0
Pt.97	8268			0	0	1	0	1	1	1	1	1	1
Pt.98	8828			1	1	1	0	0	1	0	1	0	1
Pt.99	9375			0	0	0	1	0	1	1	1	0	0
Pt.100	9643			1	1	1	0	0	1	1	1	1	0
Pt.101	9855			0	1	1	0	0	1	1	1	1	1
Pt.102	13410			0	0	1	0	0	0	0	1	1	1
Pt.103	16480			0	1	1	0	0	0	1	1	0	0
Pt.104	22540			0	1	0	0	1	0	1	1	0	0
Pt.105	25910			0	1	1	1	0	1	0	1	1	0
Pt.106	40120			1	1	1	1	1	1	1	1	1	1
Pt.107	45870			0	1	1	1	0	1	0	1	1	0

Supplemental Figure 1

E

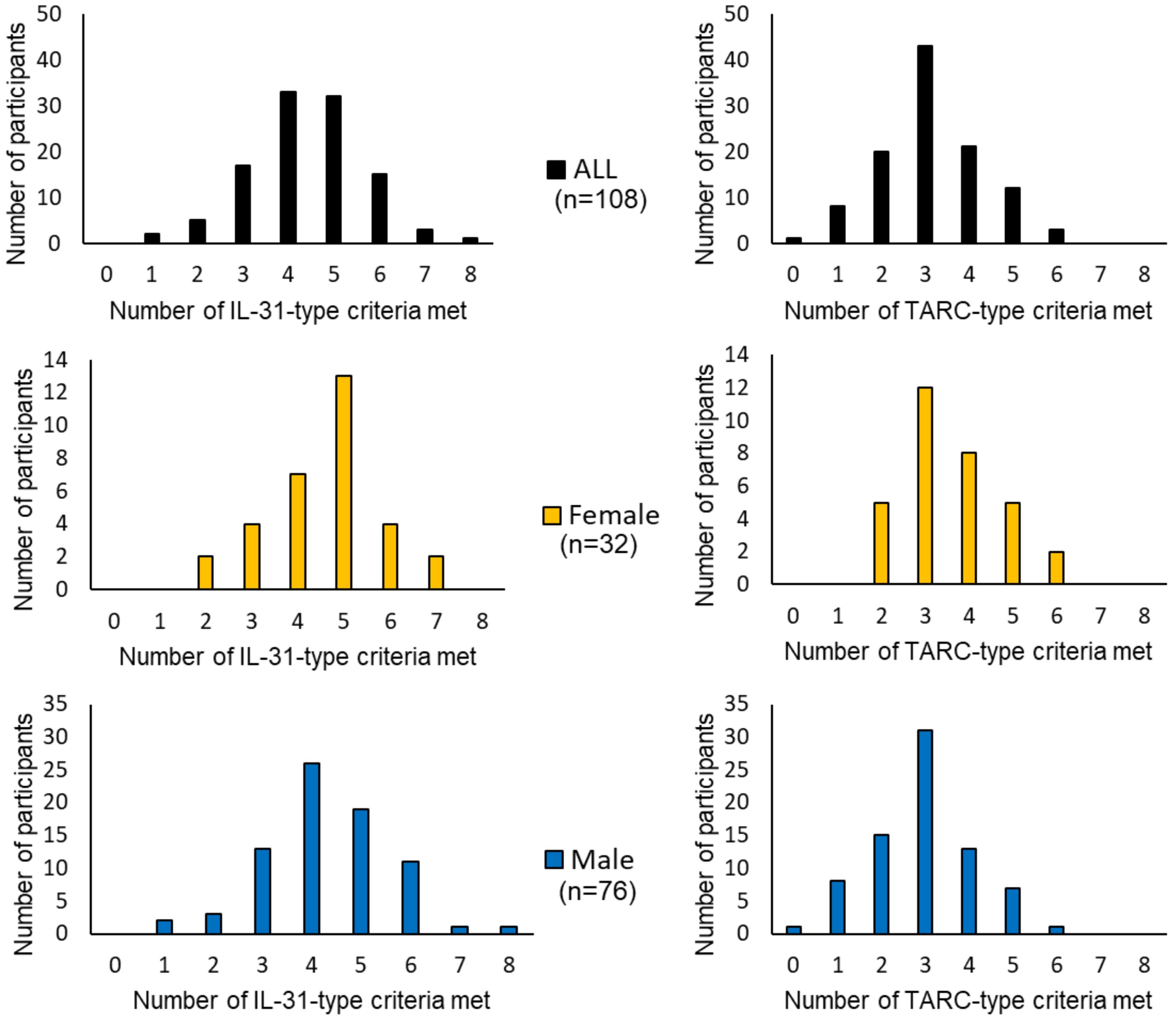

F

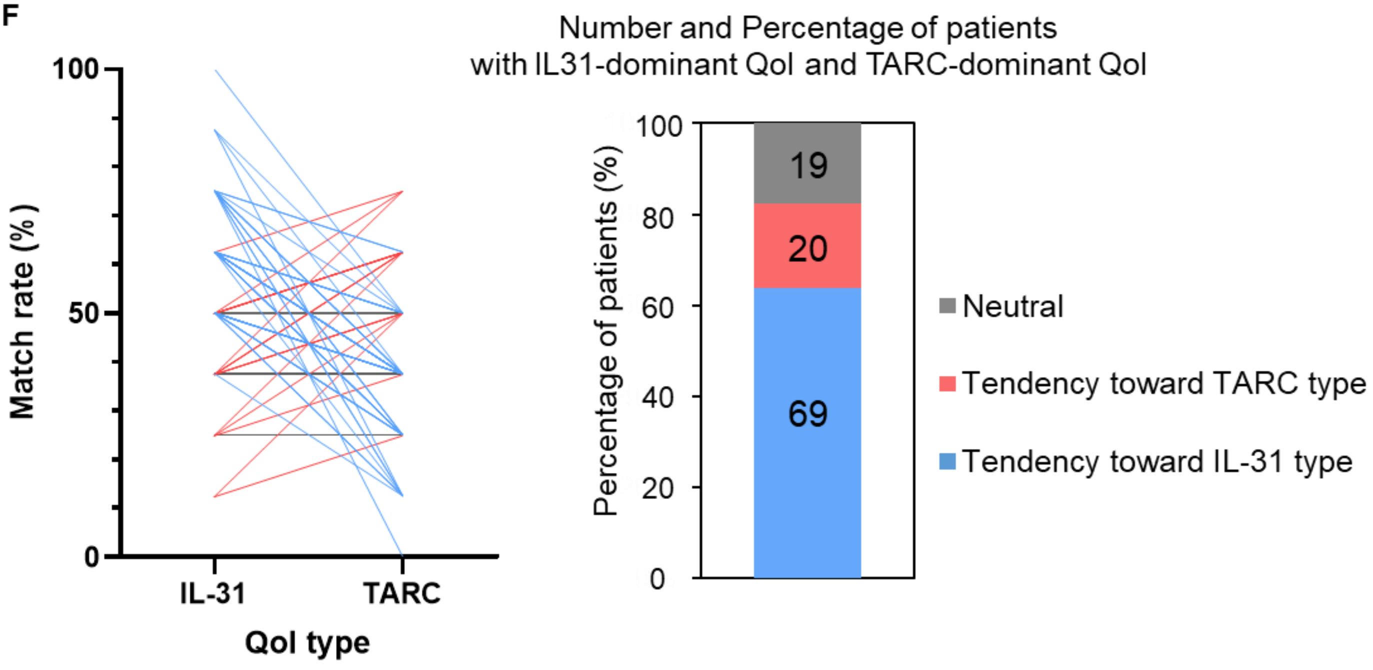

Supplemental Table 1

Supplemental Table 1 Match rate for QOI (%)

		IL-31-type		TARC-type	
Mean ± SD (Median)	All [n=108]	54.9 ± 15.9 (50.0)		39.2 ± 15.4 (37.5)	
	Female [n=32]	57.4 ± 15.5 (62.5)	ns	44.9 ± 14.1 (37.5)	*
	Male [n=76]	53.8 ± 16.1 (50.0)		36.8 ± 14.8 (37.5)	

Differences in agreement rates between males and females for each type were evaluated using the Mann–Whitney U test. The asterisk indicates statistical significance (*P* = .016).

Supplemental Figure 2

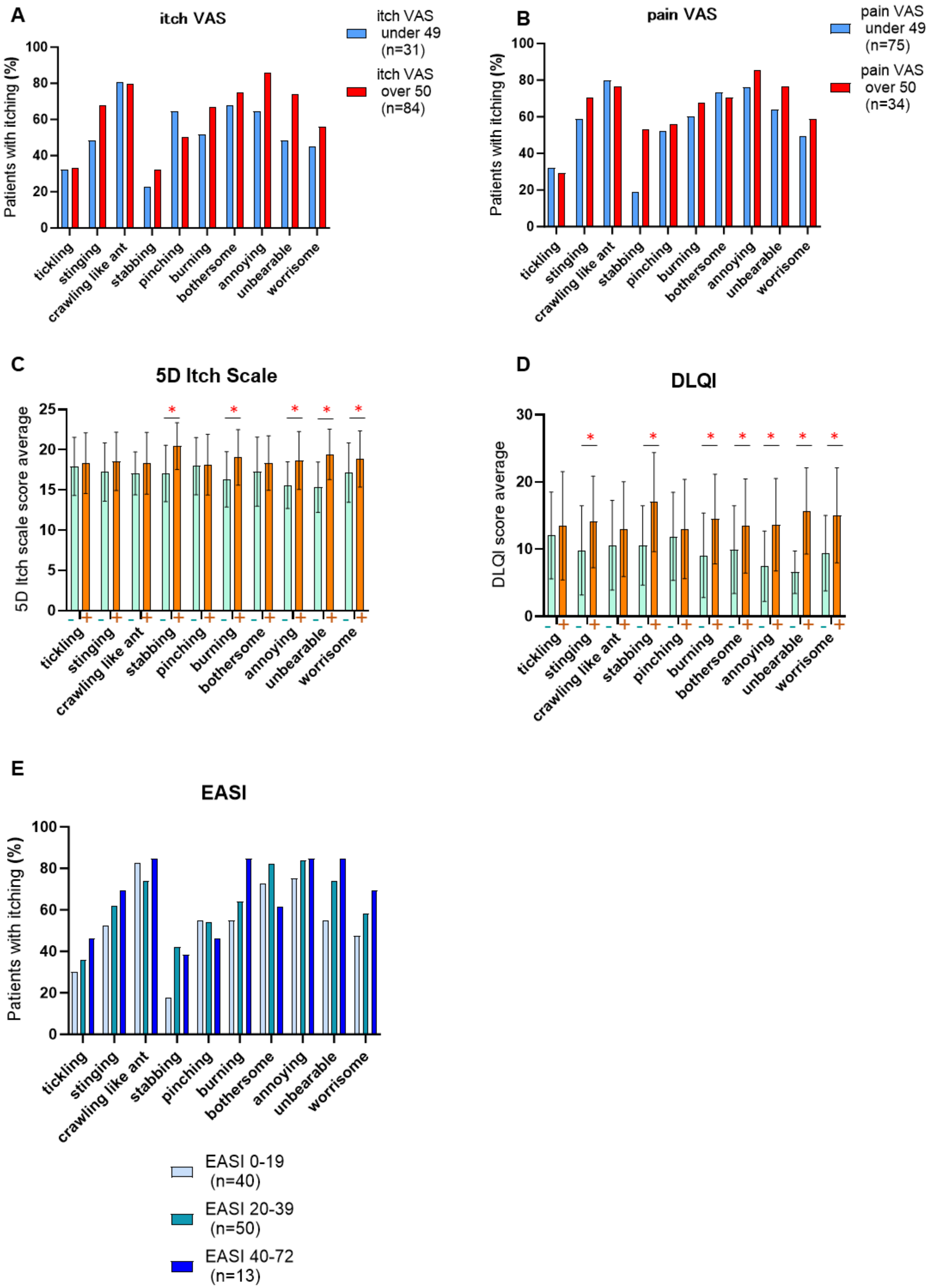

Supplemental Figure 3

A

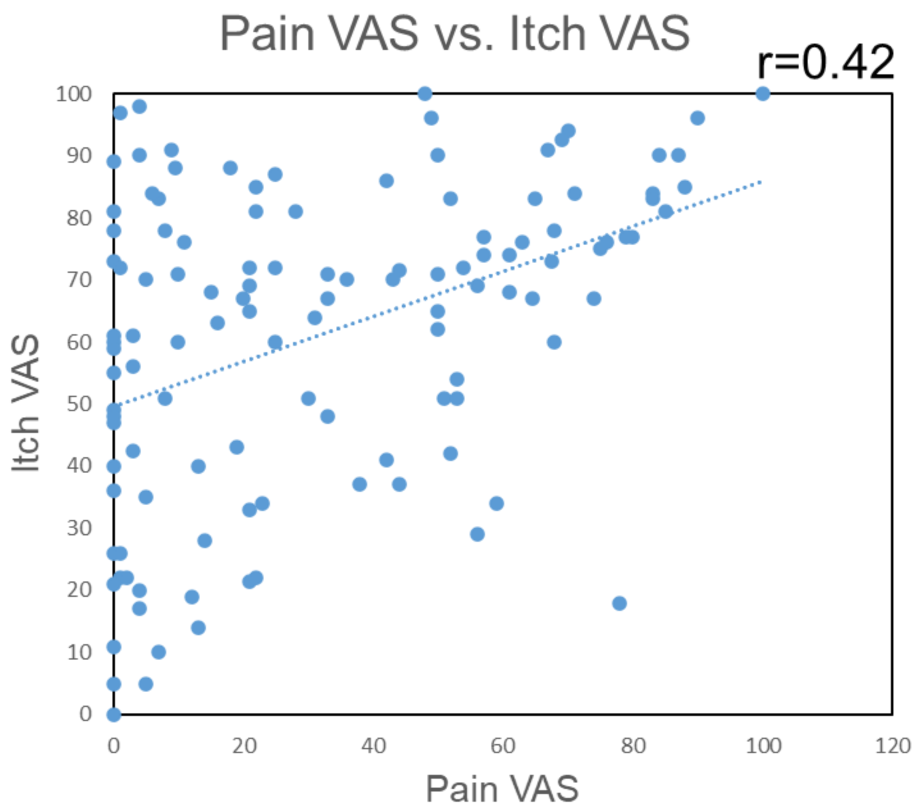

B

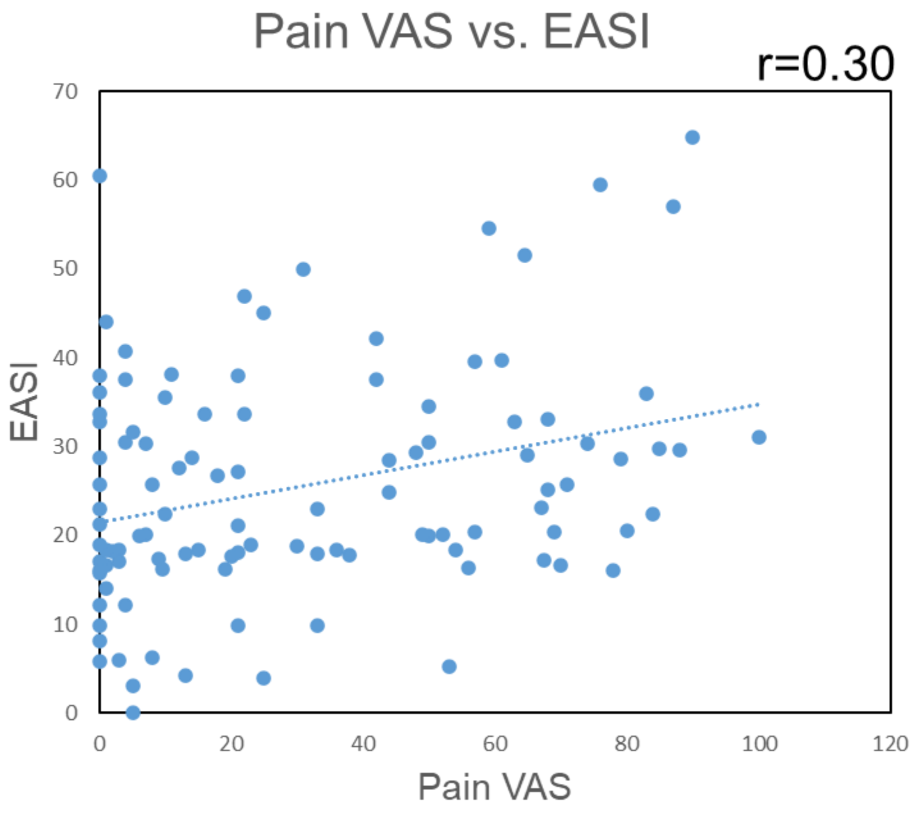

Supplemental Figure 4

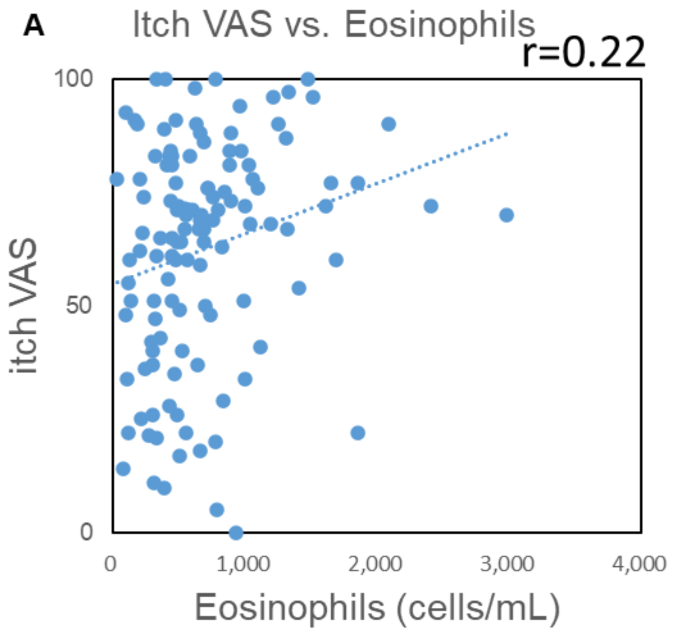

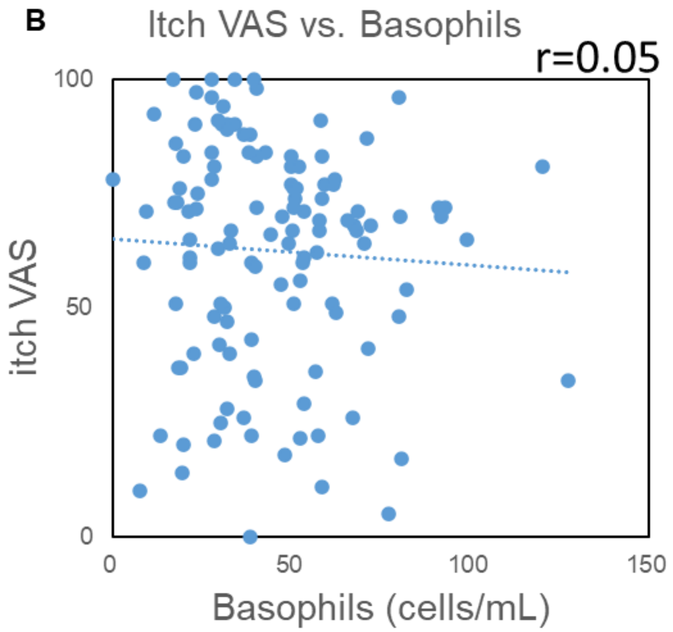

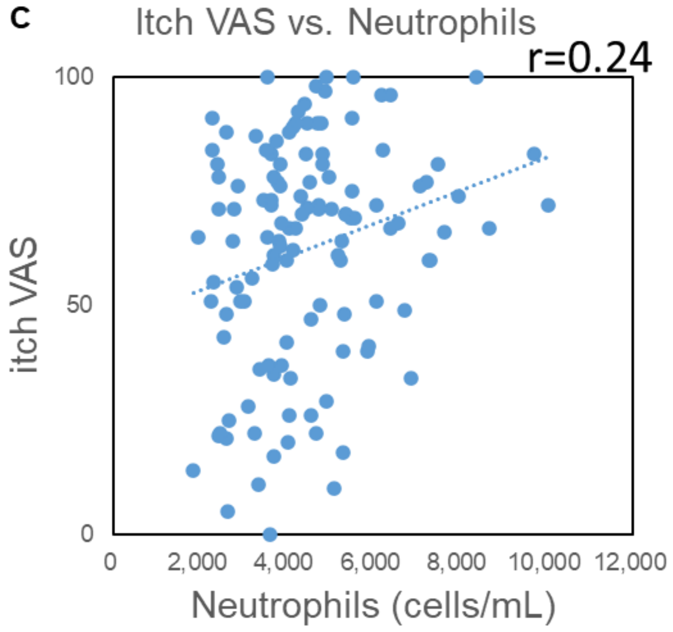

Supplement: Supplementary Figures [file mmc2.pdf]
